# Peer-led digital health lifestyle intervention for a low-income community at risk for cardiovascular diseases (MYCardio-PEER): a quasi-experimental study protocol

**DOI:** 10.1017/S1463423625000192

**Published:** 2025-03-03

**Authors:** Geok Pei Lim, Jamuna Rani Appalasamy, Badariah Ahmad, Kia Fatt Quek, Shazwani Shaharuddin, Amutha Ramadas

**Affiliations:** 1 Jeffrey Cheah School of Medicine and Health Sciences, Monash University Malaysia, Malaysia; 2 School of Pharmacy, Monash University Malaysia, Malaysia

**Keywords:** cardiovascular disease, community, lifestyle intervention, peer-led, peer support, primary prevention

## Abstract

**Background:**

Cardiovascular disease (CVD) poses a substantial global health burden, necessitating effective and scalable interventions for primary prevention. Despite the increasing recognition of peer-based interventions in managing chronic diseases, their application in CVD prevention still needs to be explored.

**Aims:**

We describe the protocol of a quasi-experiment to evaluate the effectiveness of a peer-led digital health lifestyle intervention, MYCardio-PEER, for a low-income community at risk for CVD. This study aims to assess the effectiveness of MYCardio-PEER in improving the participants’ knowledge, lifestyle behaviours and biomarkers related to CVD. Secondarily, we aim to assess the adherence and satisfaction of participants towards MYCardio-PEER.

**Methods:**

A minimum total sample of 68 low-income community members at risk for CVD will be recruited and allocated either to the control group or the intervention group. Participants in the control group will receive standard lifestyle advice and printed materials for CVD prevention, while the intervention group will participate in the 8-week MYCardio-PEER intervention program. The participants will be assessed at Week 0 (baseline), Week 8 (post-intervention) and Week 20 (post-follow-up).

**Discussion:**

We anticipate a net improvement in CVD risk score, besides investigating the effectiveness of the intervention program on CVD-related knowledge, biomarkers, and diet and lifestyle behaviours. The successful outcome of this study is essential for various healthcare professionals and stakeholders to implement population-based, cost-effective, and accessible interventions in reducing CVD prevalence in the country.

**Trial registration**: ClinicalTrials.gov (NCT06408493)

## Introduction

Cardiovascular disease (CVD) has been identified as the primary cause of mortality worldwide (Vaduganathan et al., 2022), exhibiting a higher prevalence in low-income countries (LICs) with 7.1 cases per 1000 person-years, and middle-income countries (MICs) with 6.8 cases per 1000 person-years compared to High-Income Countries (HICs) with 4.3 cases per 1000 person-years (Dagenais et al., 2020; Vaduganathan et al., 2022). In Malaysia, CVD also stands as the major contributor to the premature mortality rate, with ischaemic heart disease as the principal cause of death, which recorded 20 322 deaths (16.1%) in 2022 (DOSM, 2020a). To address this pressing issue, the Malaysian government has been actively promoting a healthy lifestyle using advertisements and awareness programs to educate the public and patients about the significance of being aware of their CVD risks (MOH, 2017). Despite these efforts, the prevalence of CVD risk factors, including hypertension, dyslipidaemia, and diabetes, continues to rise among Malaysians, as reported in the recent Malaysian Health and Morbidity Survey (NIH, 2019).

Lifestyle risk factors such as unhealthy diet, physical inactivity, tobacco use, and alcohol consumption significantly contribute to the escalating prevalence of CVD (CDC, 2021). Consequently, lifestyle modifications remain pivotal in the primary prevention of CVD, especially among high-risk populations (Rippe & Angelopoulos, 2019; Kaminsky et al., 2022). While lifestyle interventions targeting these adverse health behaviours have shown promise, their implementation in community settings faces challenges such as workforce limitations, cost considerations, program acceptability, and cultural adaptation (Milat et al., 2014). Furthermore, healthcare professionals may lack the necessary skills, time, and socio-cultural understanding to provide ongoing support and empowerment for behavioural change in managing CVD risk factors (Moudatsou et al., 2020). Therefore, the role of social environment and support in facilitating practical behaviour change interventions within the community is crucial (Latkin & Knowlton, 2015).

Peer support, where individuals with similar backgrounds and experiences provide support, is vital in promoting and maintaining complex health behaviours and managing non-communicable diseases (NCDs) (Fisher et al., 2017; Fortuna et al., 2019). Peers offer a sense of community and ongoing emotional support, enhancing motivation to overcome barriers to behaviour change (Fisher et al., 2023). Recent advances in peer support have expanded its functions to include assistance in daily disease management and acting as a link to clinical and community resources (Evans et al., 2021). A recent systematic review evaluated and reported favourable effects of peer-led interventions on the primary prevention of CVD (Lim et al., 2024). However, peer-led intervention as a viable community approach to prevent CVD still needs to be explored among the low-income communities in Malaysia.

Digital technologies, particularly those using bite-sized videos and infographics, offer a promising solution for ensuring the scalability and accessibility of peer-led interventions. Digital technologies have emerged as powerful tools in healthcare, offering innovative ways to deliver interventions and engage individuals in managing their health (Stoumpos et al., 2023). The widespread use of smartphones and internet access has created unprecedented opportunities to reach populations traditionally underserved by organised healthcare services (Xiong et al., 2023). Videos, for example, can deliver information in a visually engaging and interactive format, making complex concepts easier to understand and remember (Tuong et al., 2014). By incorporating evidence-based guidelines, these videos can educate individuals about modifiable risk factors for CVD and provide practical strategies and step-by-step guidance on adopting healthier habits, thus empowering individuals to take control of their cardiovascular health.

Despite the benefits of peer support and digital technologies, no interventional study in Malaysia integrates both, and none of the CVD primary prevention interventions have focused on low-income communities (Raman et al., 2024). There is a clear research gap regarding the application of peer-led digital health lifestyle interventions for managing CVD risk, particularly among the low-income community in Malaysia. Hence, we have designed a quasi-experimental study to evaluate the effectiveness of a peer-led digital health lifestyle intervention, MYCardio-PEER, for a low-income community at risk for CVD. This study aims to assess the effectiveness of MYCardio-PEER in improving the participants’ knowledge, lifestyle behaviours and biomarkers related to CVD, and to assess the adherence and satisfaction of participants towards MYCardio-PEER.

## Materials & methods

### Study design

This study will adopt a quasi-experimental study design. As this intervention study relies on peer support, a quasi-experiment is considered the most appropriate study design due to the practical barrier for randomisation, including participants’ locality and peer connectivity (White & Sabarwal, 2014). The study protocol was developed in accordance with the Transparent Reporting of Evaluations with Nonrandomized Designs (TREND) statement and checklist as presented in Supplementary Table 1 (Des Jarlais et al., 2004). This study has been registered on ClinicalTrials.gov (NCT06408493).

### Study flow

**Figure 1.**
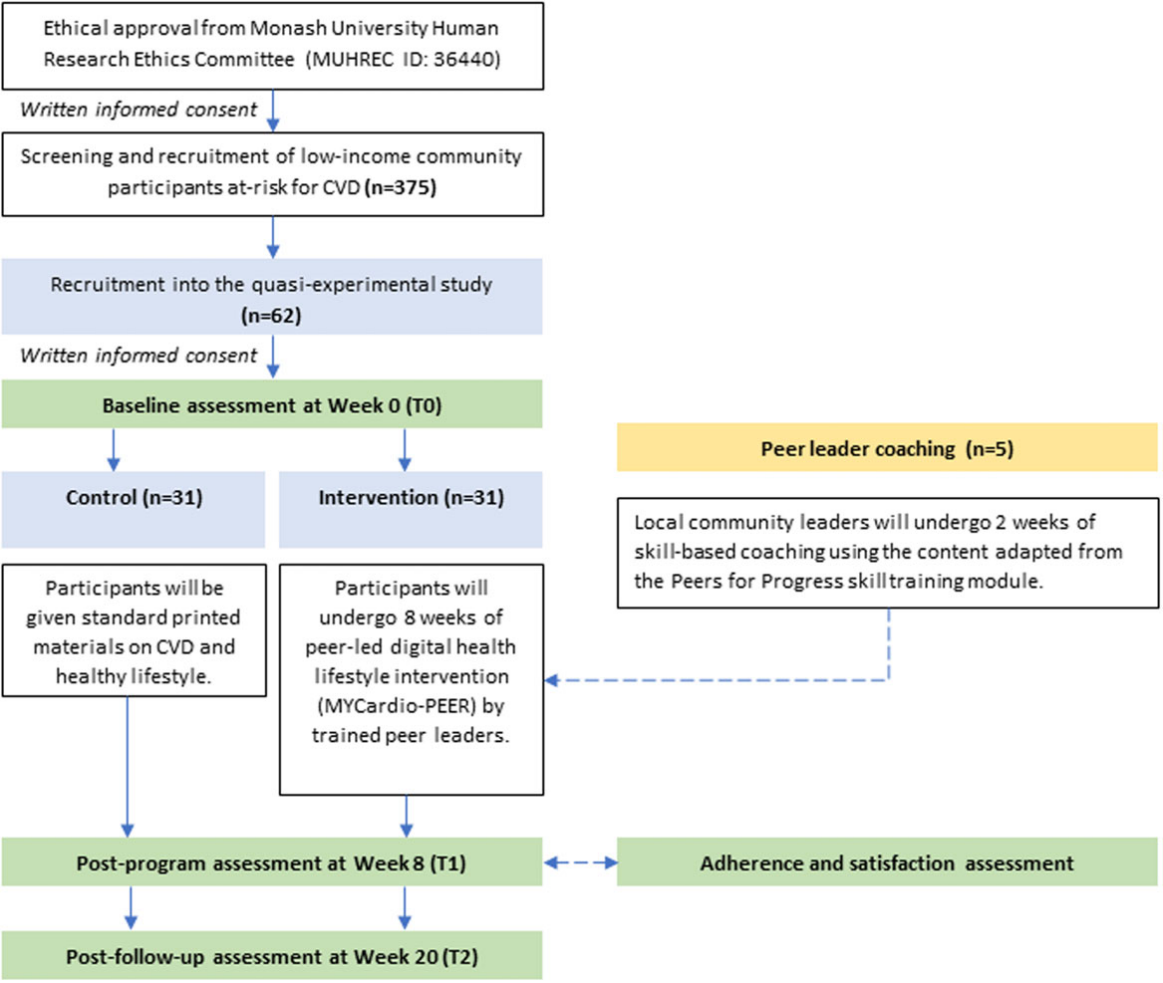
Flow chart of the proposed study.

This study is to be completed in 20 weeks, comprising an initial eight-week core intervention aimed at promoting health behaviour adoption (Bajer et al., 2019), followed by a 12-week follow-up period post-intervention to evaluate its sustainability (Khouja et al., 2020). Figure 1 depicts the flow chart of the proposed study. Local community members will be invited to medical camps that will be used as screening and recruitment camps for the quasi-experiment study. Upon completion of the baseline assessment at Week 0 (T0), the participants will be assigned into two groups: intervention or control. The intervention will be conducted for eight weeks, followed by the post-intervention assessment at Week 8 (T1). The post-follow-up assessment will be conducted at Week 20 (T2), whereby there will be no active intervention within the 12 weeks.

The intervention lasts for eight weeks, allowing time for participants to acquire CVD-related knowledge and self-regulation skills with peer support, thus improving the proximal outcome in terms of CVD risk factors and lifestyle behaviours. As for the 12 weeks of no intervention period, it is necessary to evaluate whether participants maintain the adopted behaviours autonomously and whether the initial intervention effects persist over time in improving the distal outcome of cardiovascular health (Ryan, 2009; Gold et al., 2021). The participants’ adherence and satisfaction with the intervention program will also be assessed at T2. Peer leader coaching based on the Peer for Progress training module (Peer for Progress, 2021) will be provided to the selected community leaders before the intervention.

### Setting

Our study will be conducted in Kulim, Kedah, Malaysia, in collaboration with the Kamakshi Welfare and Social Association (KWSA). Kedah, a state in the country’s northern region, is not only identified as one of the economically challenged states (DOSM, 2020b). Its population also has the highest CVD risk score based on region-specific analysis (Che Nawi et al., 2022). Furthermore, Kulim is among the administrative districts in Kedah that exhibit a high poverty rate, which has been on the rise from 2019 to 2022 (DOSM, 2023).

In conjunction, KWSA located in Kulim, Kedah, actively engages in community outreach initiatives that primarily assist single, marginal, or economically disadvantaged individuals by providing career guidance, financial assistance, nutritious foods and essential household items (PKDS, n.d.). Within this context, KWSA has good acquaintances with the community’s needs. By partnering with KWSA, this study can leverage their deep understanding of the local low-income community and their established presence in the area, thus ensuring that the intervention is tailored to the unique circumstances and preferences of the target population, maximising its potential impact and effectiveness. In return, the findings from the current study can contribute to the implementation of personalised health interventions within this community.

### Participants

#### Sampling and recruitment

Convenience sampling complemented by snowball sampling techniques will be employed for this study. Firstly, we collaborated with KWSA as the grassroot organisation to disseminate the information about the medical screening camp to the community members in their contact database list. The medical screening camp is part of a larger study to identify individuals at moderate to high risk of CVD. Secondarily, participants in the screening camp will be encouraged to extend invitations to other potential participants through personal recommendations. This combined approach ensures our study reaches the target population by utilising established community networks and connections, making the research culturally appropriate and community-focused (Valerio et al., 2016). It also facilitates the formation of peer groups for the intervention, fostering trust and rapport, increasing engagement in peer-led activities that promote lifestyle behavioural change.

During the medical screening camp, we will obtain socio-demographic information, anthropometry measurements, clinical measures, and calculated Framingham General CVD Risk Score (FRS). By matching the eligibility criteria, explanatory statements and consent forms about the study will be provided to recruit the potential participants. Informed consent to participate in the study will be obtained before baseline data collection. The minimum age of 30 years was set to align with the applicability of the FRS, which is designed for individuals aged 30 years and older. Table 1 shows the eligibility criteria of study participants.

**Table 1.** Eligibility criteria of study participants

Inclusion	Exclusion
Mentally sound individuals of at least 30 years old	Pregnant, lactating or intend to become pregnant during the study period
Have a monthly household income of less than RM3,710 [30] or receiving government aid for low-income community	Have other pre-existing medical conditions or severe complications which could compromise the ability to adhere to the study
Literate with a fair command of the Malay language	Have been enrolled in other studies
Have a mobile device connected to the Internet	
Willing to attend eight weeks of intervention and be followed up for another 12 weeks during the maintenance phase	
Have been confirmed to have a moderate or high risk for CVD based on the Framingham General CVD Risk Score (FRS) [4]	

Peer leaders will be selected from within the same community and screened for moderate to high risk of CVD. This ensures that the leaders share relevant experiences with the target population. The selection process will include discussions and recommendations by the social welfare centre to assess candidates’ leadership and communication skills. Additionally, personal interviews will be conducted to further evaluate their suitability and commitment to supporting the program’s objectives. Candidates will also undergo training to equip them with the necessary skills to facilitate intervention activities and encourage behaviour change.

#### Sample size justification

The sample size for the quasi-experiment was calculated using the G * Power 3.1 sample size calculator software (Faul et al., 2009). Based on expected changes in the FRS with a medium effect size of 0.25, the minimum number of participants required in each intervention and control group is 28, giving this study 80% power at a significance level of 0.05 (two-tailed test). Considering the prospective nature of the study and attrition rate of 20%, a minimum number of 34 participants will be recruited into each study group.

### Participant allocation

Two groups will be under study for this quasi-experiment: the intervention group and the control group. The recruited participants will be allocated into one of the two groups based on their geographical location to ensure participants’ convenience when participating in the intervention activities. The participants in the intervention will be further divided into five subgroups, each consisting of six to seven members based on their peer connectivity. The non-random allocation considers familiarity and peer support, which are expected to lead to higher engagement levels in the intervention activities, promoting better adherence to the intervention and potentially more significant lifestyle behavioural changes.

#### Control group

Participants in the control group will receive personalised lifestyle advice from a registered nutritionist at baseline, and standard printed materials on CVD prevention and healthy lifestyle habits. The control group participants will only be contacted for re-assessment at T1 and T2. The intervention content of MYCardio-PEER and peer support will not be provided to the control group.

#### Intervention group

The participants in the intervention group will undergo the MYCardio-PEER program for eight weeks, which will be led by trained peer leaders. Participants will be added to a WhatsApp group whereby bite-sized educational videos, infographic posters, and interactive activities that cover knowledge, nutrition and lifestyle behaviours for CVD prevention will be shared. Besides that, the peer leaders will also organise physical meeting sessions at convenient locations with group members, including an exercise club and healthy food preparation demonstration.

### Intervention program

**Figure 2.**
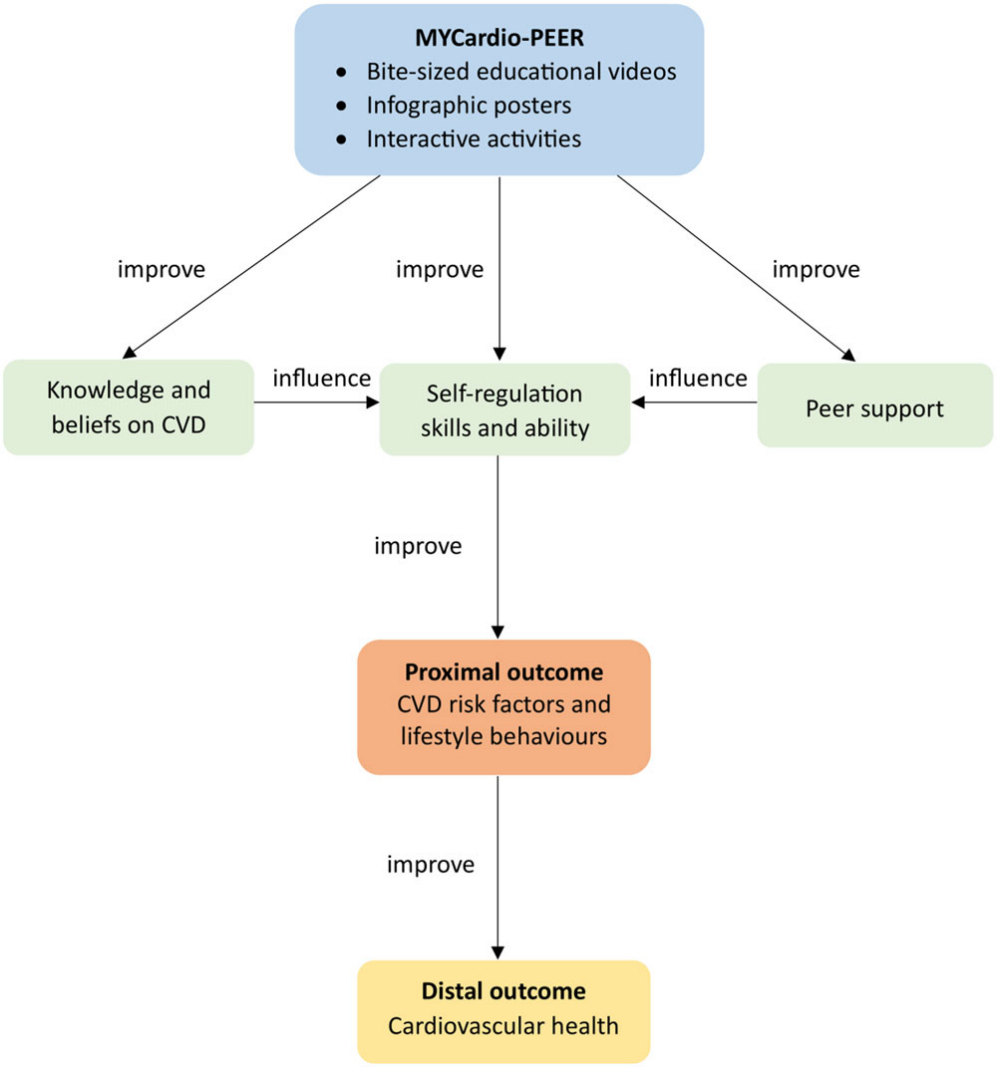
Conceptual framework of the intervention program.

The elements of the MYCardio-PEER intervention program are developed based on the current evidence on peer-led lifestyle intervention for primary prevention of CVD (Lim et al., 2024), clinical practice guidelines on primary and secondary prevention of CVD (MOH, 2017) and Malaysian Dietary Guidelines (NCCFN, 2021). The behaviour change objectives, intervention strategies and delivery methods are developed using the Integrated Theory of Health Behaviour Change (Ryan, 2009). The conceptual framework of the intervention program is presented in Fig. 2. The details of the program development will be described in subsequent publication. Briefly, MYCardio-PEER intervention is expected to improve the knowledge and beliefs related to CVD, self-regulation skills and ability, and provide peer support. This, in turn, improves CVD risk factors and lifestyle behaviours, and eventually improves cardiovascular health.

The peer leaders will distribute evidence-based bite-sized educational videos and infographic posters developed for MYCardio-PEER via a WhatsApp group chat platform each week. These multimedia materials will comprehensively address topics related to knowledge dissemination, nutritional guidance, and lifestyle modifications pertinent to CVD prevention. The general topics and objectives of digital content are presented in Table 2. Within the week, peer leaders will also introduce interactive activities through the group chat or organise face-to-face sessions to enhance learning and practice behaviours. In the group chat, participants will be encouraged to pose questions, challenges, or experience sharing relevant to reducing CVD risk.

**Table 2.** Topics and objectives of MYCardio-PEER intervention by week

Week	Topic	Objectives
1	Are you at risk of heart disease?	Identify the modifiable risk factors associated with CVD
2	Say YES to physical activity	Encourage the adoption of an active lifestyle
3	How to maintain a healthy weight?	Achieve and maintain a healthy body weight
4	Control your blood pressure	Obtain practical diet and lifestyle strategies for controlling blood pressure
5	Keep your cholesterol in check	Obtain practical diet and lifestyle strategies for maintaining healthy cholesterol levels
6	The not-so-sweet truths	Obtain practical diet and lifestyle strategies for maintaining healthy blood glucose levels
7	Enjoy living smoke-&-stress-free	Encourage a smoke-free lifestyle Enhance stress management
8	Practical tips for heart-healthy meals	Encourage the adoption of healthy eating habits that align with dietary guidelines for cardiovascular health

### Outcome measures

The primary outcome of this study is the change in FRS, which is calculated based on age, sex, levels of high-density lipoprotein cholesterol (HDL-C) and total cholesterol, systolic blood pressure (SBP), smoking habit and the presence of diabetes. This risk calculator has been validated in the Malaysian local population and is recommended for assessing and managing the global risk of individuals in primary prevention of cardiovascular disease (CVD) (MOH, 2017).

The secondary outcomes include changes in body weight, body mass index (BMI), waist circumference, hip circumference, fasting blood glucose (FBG), low-density lipoprotein cholesterol (LDL-C), triglycerides (TG) levels, diastolic blood pressure (DBP), Medication Taking and Understanding Self-efficacy (MUSE), attitudes and beliefs about CVD, physical activity level, lifestyle habits (smoking, alcohol consumption and sleep duration), stress level, edible oil use and dietary intakes.

A process evaluation will be conducted at T1 among the intervention participants to assess their adherence to the program and satisfaction towards content and peer leadership.

### Data collection

Study researchers will administer a pretested, structured questionnaire in both Malay and English language to collect data of the outcome measures at T0, T1 and T2. Sociodemographic data and medical history will be collected at T0 only. At T1, the program feedback form and questionnaire on content satisfaction will be administered among the intervention participants.

#### Clinical measures

The levels of total cholesterol, HDL-C, LDL-C and TG will be measured using the CardioChek PA analyser (PTS Diagnostics, USA). Blood pressure will be measured using the automatic blood pressure monitor (HEM-7120, Omron Healthcare, Japan). Fasting blood glucose will be assessed using the GlucoSure Autocode metre (Apex Biotechnology Corp, Taiwan). All measurements will be taken based on the standard procedures. Blood pressure will be measured twice to get the average readings.

#### Anthropometric measures

For anthropometric assessments, participants will be asked to attend in light clothing with shoes removed. Body height and weight will be assessed using the Seca 777 digital column scale (Seca, Germany). Waist and hip circumference will be measured using the Seca measuring tape 201 (Seca, Germany). All measurements will be taken based on the standard procedures and measured twice to get the average readings. The BMI will then be calculated and recorded.

#### Knowledge and self-efficacy

The CVD-related knowledge will be assessed using the Attitudes and Beliefs about Cardiovascular Disease (ABCD) risk questionnaire which has been validated in the Malaysian population (Woringer et al., 2017; Mat Said et al., 2022). This questionnaire may assess the accuracy of perceived CVD risk, overall knowledge of CVD, and the intention to modify behaviour concerning diet and exercise.

The Medication Understanding and Taking Self-Efficacy (MUSE) scale will be used to assess medication-taking and understanding self-efficacy, as medication adherence can reduce the risk of developing CVD (Cameron et al., 2010; Appalasamy et al., 2019). This scale assesses various aspects of medication management, including understanding instructions, remembering to take medications, and integrating medication use into daily routines.

#### Lifestyle behaviours

Physical activity will be assessed using the International Physical Activity Questionnaire (IPAQ) (Craig et al., 2003; Chu & Moy, 2015). The IPAQ provides a comprehensive overview of a participant’s physical activity patterns, allowing for the categorisation of individuals into different activity levels and assessment of sedentary behaviour. The Perceived Stress Scale (PSS-10) (Cohen et al., 1983; Al-Dubai et al., 2014) will be used to assess the perceived stress level of participants as chronic stress can contribute to the development and exacerbation of CVD risk factors (Kivimäki & Steptoe, 2018). Other information on lifestyle behaviours including smoking habits, alcohol consumption and sleep duration will also be obtained from participants as these behaviours can influence CVD risk factors (Kaminsky et al., 2022).

#### Dietary intake

The dietary intake will be assessed using the short food frequency questionnaire, while nutrient intake will be assessed using 24-hour dietary recall (Johansson, 2014). For the dietary recall, the participants will be asked about details of the foods and beverages consumed in the past 24 h, including the types of food, cooking methods, estimated portion size and brands. The recalls will be made for weekdays and weekends using the multiple-pass approach (Gibson, Charrondiere & Bell, 2017). A food album (Suzana et al., 2015) and household measurement tools will facilitate food portion estimation.

#### Process evaluation

The peer attendance will assess the adherence to the program to each peer session. The program feedback will be recorded in a self-administered questionnaire post-program (T1), assessing the participants’ satisfaction with the content satisfaction and peer leaders. We will use a five-factor instrument to determine content satisfaction: time and place for peer session, module objective, module content, module structure, and module relevance. In addition, peer leadership will be assessed with a three-factor instrument: peer interactions, peer leader readiness, and peer leader knowledge. The responses will be recorded using a Likert scale with scores ranging from 1 (strongly disagree) to 5 (strongly agree). The sum of the responses for each domain (content and peer leadership) reflects the participants’ satisfaction, with higher scores indicating a higher level of satisfaction (Mahadzir et al., 2020).

### Statistical analysis

Data analyses will be conducted using the Statistical Package for the Social Sciences (SPSS) version 28.0 (IBM Corp., USA). Nutrient intakes from 24-hour dietary recalls will be analysed with Nutritionist Pro software (Axxya Systems, USA) to yield total energy, macronutrient and micronutrient intakes, then transferred to SPSS for analysis. Other data obtained from the assessment will be directly entered and analysed using the SPSS software. Descriptive statistics will be calculated for each variable. Continuous variables will be expressed as a mean with standard deviation (SD) or median with interquartile range. Frequencies and percentages will be obtained for categorical variables. Independent t-test or equivalent will determine differences between the study groups for continuous variables. McNemar and Chi-Square tests will be performed for similar comparisons for categorical variables. Comparison of changes in outcome measures at T0, T1 and T2 between study groups will be analysed using two-way repeated measures analysis of covariance (ANCOVA) to adjust for any baseline differences in key variables. Correlation analyses will be performed between satisfaction and adherence, with changes in outcome measures at T1 and T2.

### Ethical consideration

Ethical clearance for this study has been obtained from the Monash University Human Research Ethics Committee (MUHREC ID: 36 440). Informed consent will be obtained from each study participant. Potential risks, discomforts, and inconveniences to enrol in the study are minimal. Should participants feel uncomfortable with the study procedures, they are free to withdraw from the study at any point. All collected data will remain strictly confidential, solely for research purposes, and will not be disclosed.

## Discussion

This is a novel peer-led digital health lifestyle intervention in a low-income community at risk of CVD to improve CVD-related knowledge, biomarkers and lifestyle behaviours. The local existing guidelines are used to guide the development of the intervention program. The developed program contents are validated by healthcare professionals and local community members to ensure cultural relevance and appropriateness. We anticipate a net improvement of CVD risk score based on FRS, besides investigating the impact of the intervention program on the CVD-related knowledge, biomarkers, and diet and lifestyle behaviours.

This community-driven and culturally-sensitive intervention is centred on the low-income community so that preventive healthcare can be reached equally for the underserved population and reduce the prevalence of CVD. Besides providing theory and evidence-based digital educational contents that are easily accessible, peer leaders initiate interactive activities that enhance healthy lifestyle adoption. Peer group communication further enhances social facilitation for sustaining healthy lifestyle behaviours, leaving a more significant impact on improving cardiovascular health.

Besides the effectiveness in improving outcome measures, process evaluation forms part of the outcome evaluation of this intervention study. By assessing the participants’ adherence and satisfaction with the program alongside qualitative feedback, this study enables us to interpret the program’s fidelity and feasibility and identify potential enhancers or barriers for future implementation. Using the real-world study design with the local context, MYCardio-PEER can be readily translated into practice if positive findings are reaped from this study.

While our study possesses several strengths, there are inherent limitations to consider. First, the non-random allocation of participants into the intervention and control groups based on geographical location may introduce selection bias. Participants in different geographic areas may have varying characteristics that could affect the outcomes. Furthermore, reliance on self-reported data for variables such as physical activity, stress levels, and lifestyle behaviours may introduce response bias and measurement error. Finally, the quasi-experimental nature of the study limits our ability to control for all potential confounding variables, which may impact the internal validity of the findings. Despite these limitations, our study aims to provide valuable insights into the effectiveness of the intervention on cardiovascular health outcomes within our target population.

## Supporting information

Lim et al. supplementary material Lim et al. supplementary material

## Supplementary material

To view supplementary material for this article, please visit https://doi.org/10.1017/S1463423625000192
